# Hematological Adaptation Without Differences in Systemic Inflammatory Indices in Cyanotic and Acyanotic Congenital Heart Disease

**DOI:** 10.3390/jcm15093274

**Published:** 2026-04-25

**Authors:** Damla Erden, Ahmet Bulent Polat, Naile Fevziye Misirlioglu, Hafize Uzun

**Affiliations:** 1 Department of Pediatric Cardiology, Faculty of Medicine, Istanbul Atlas University, Istanbul 34403, Türkiye; 2 Department of Pediatric Cardiovascular Surgery, Faculty of Medicine, Istanbul Atlas University, Istanbul 34403, Türkiye; 3 Department of Medical Biochemistry, Faculty of Medicine, Istanbul Atlas University, Istanbul 34403, Türkiye

**Keywords:** congenital heart disease, cyanosis, hematological adaptation, systemic inflammation, neutrophil-to-lymphocyte ratio, systemic immune–inflammation index

## Abstract

**Background**: Chronic hypoxemia in cyanotic congenital heart disease triggers well-recognized hematological adaptation; however, whether hypoxemia also drives systemic inflammatory activation remains uncertain. This study aimed to evaluate hematological parameters and inflammatory indices in cyanotic and acyanotic congenital heart disease (CHD) to better characterize the relationship between hypoxemia and systemic inflammatory status. **Methods**: In this single-center retrospective study, 260 children with congenital heart disease were classified as cyanotic (*n* = 158) or acyanotic (*n* = 102). Preoperative clinical data and laboratory parameters were analyzed, including oxygen saturation, hemoglobin, hematocrit, leukocyte indices, C-reactive protein (CRP), and procalcitonin (PCT). Inflammatory indices derived from complete blood counts were calculated, including the neutrophil-to-lymphocyte ratio (NLR), platelet-to-lymphocyte ratio (PLR), and systemic immune–inflammation index (SII). **Results**: Oxygen saturation was significantly lower in cyanotic patients than in acyanotic patients (75 ± 9% vs. 95 ± 4%, *p* < 0.001). Consistent with hypoxemia-driven hematological adaptation, hemoglobin and hematocrit levels were significantly higher in the cyanotic group (16.1 ± 2.9 g/dL vs. 13.1 ± 2.0 g/dL and 50.8 ± 9.7% vs. 39.7 ± 5.5%, respectively; *p* < 0.001). In contrast, inflammatory indices (NLR, PLR, and SII) were similar between cyanotic and acyanotic patients, and no significant associations were observed between oxygen saturation and these inflammatory indices. **Conclusions**: While cyanotic congenital heart disease demonstrates marked hematological adaptation secondary to chronic hypoxemia, systemic inflammatory indices appear similar in cyanotic and acyanotic patients. These findings suggest a relative dissociation between hypoxemia-driven hematological responses and the evaluated systemic inflammatory indices, indicating that inflammatory burden in congenital heart disease may not be solely explained by cyanosis and may reflect additional underlying mechanisms not captured by these markers.

## 1. Introduction

Congenital heart disease (CHD) is the most common congenital anomaly worldwide and represents a heterogeneous group of structural cardiac malformations with diverse hemodynamic consequences [1,2,3,4]. Based on systemic oxygenation, CHD is broadly classified into acyanotic and cyanotic forms [1,2]. Acyanotic defects, such as atrial septal defect and ventricular septal defect, typically involve left-to-right shunting with preserved systemic oxygenation. In contrast, cyanotic defects, including tetralogy of Fallot, are characterized by right-to-left shunting that results in chronic systemic hypoxemia [1,2]. This persistent hypoxemic state induces several physiological adaptations, most notably increased erythropoiesis and elevated hemoglobin and hematocrit levels as compensatory mechanisms to maintain oxygen delivery to tissues [1,2].

In patients with cyanotic congenital heart disease, chronic exposure to hypoxemia induces compensatory hematological adaptations aimed at enhancing oxygen-carrying capacity. This response is typically reflected by increased hemoglobin and hematocrit levels, representing a well-established physiological mechanism to maintain tissue oxygen delivery [5,6]. However, whether chronic hypoxemia also promotes systemic inflammatory activation in this population remains uncertain.

In recent years, several inflammatory indices derived from routine peripheral blood counts, including the neutrophil-to-lymphocyte ratio (NLR), platelet-to-lymphocyte ratio (PLR), and systemic immune–inflammation index (SII), have emerged as accessible and practical markers of systemic inflammatory status [7,8,9,10,11,12]. Although these biomarkers have been increasingly investigated in various cardiovascular conditions, data regarding their baseline levels in pediatric congenital heart disease, particularly in relation to cyanotic and acyanotic phenotypes, remain limited.

Therefore, the present study aimed to evaluate the relationship between hematological adaptation parameters and systemic inflammatory indices (NLR, PLR, and SII) in children with cyanotic and acyanotic congenital heart disease, to better characterize the inflammatory profile associated with these conditions and to assess their potential clinical relevance.

## 2. Materials and Methods

### 2.1. Study Design

This retrospective cross-sectional study was conducted at Istanbul Atlas University Hospital and included pediatric patients who underwent surgical treatment for congenital heart disease between 2022 and 2025. A total of 260 children diagnosed with congenital heart disease were enrolled in the study.

The study protocol was approved by the Institutional Non-Interventional Research Ethics Committee of Istanbul Atlas University (approval date: 26 January 2026; decision no: 51), and the study was conducted in accordance with the principles of the Declaration of Helsinki.

### 2.2. Patient Classification

The study population was classified into two main groups according to their systemic oxygenation status and underlying hemodynamic characteristics.

The cyanotic group (*n* = 158) consisted of patients with congenital heart defects associated with chronic systemic hypoxemia and right-to-left shunting. This group included patients with tetralogy of Fallot, single-ventricle physiology, and other complex cyanotic malformations.

The acyanotic group (*n* = 102) included patients with congenital heart defects characterized by left-to-right shunting or obstructive lesions with preserved systemic oxygen saturation, such as atrial septal defect, ventricular septal defect, and other non-cyanotic lesions.

### 2.3. Study Population, Inclusion and Exclusion Criteria

#### 2.3.1. Sample Size and Power Analysis

The required sample size was determined using a formal power analysis based on previous studies evaluating inflammatory indices in congenital heart disease. To achieve a statistical power of 80% at a 95% confidence level with an effect size of 0.40, a minimum sample size of 200 patients was required. To further increase statistical robustness and account for potential data variability, a total of 260 eligible patients (158 cyanotic and 102 acyanotic) were ultimately included in the final analysis.

As this was a retrospective study, no scales or questionnaires were administered. Clinical and laboratory data were systematically retrieved from the Hospital Information Management System and the institutional electronic laboratory database. All patient data were anonymized prior to analysis to ensure confidentiality.

To ensure a homogeneous and clinically relevant study population, the following inclusion and exclusion criteria were applied:

#### 2.3.2. Inclusion Criteria

1. Pediatric Population: Patients aged between 0 and 18 years.
2. Confirmed Diagnosis: A definitive diagnosis of CHD, classified as either cyanotic or acyanotic based on clinical, echocardiographic, or angiographic findings.
3. Comprehensive Laboratory Data: Availability of complete preoperative hematological and biochemical profiles, including hemoglobin (Hb), hematocrit (Hct), white blood cell (WBC) count, absolute neutrophil and lymphocyte counts, platelet count, C-reactive protein (CRP), and procalcitonin (PCT).

#### 2.3.3. Exclusion Criteria

To eliminate potential confounders of systemic inflammation and hematological indices, patients meeting any of the following criteria were excluded:

1. Acute or Chronic Inflammatory States: Presence of active systemic infection, sepsis, or known autoimmune/inflammatory disorders at the time of surgery.
2. Primary Hematological Disorders: Patients with hereditary or acquired hematological conditions, such as thalassemia, sickle cell anemia, leukemia, or other bone marrow disorders.
3. Organ Dysfunction: Co-existing chronic hepatic, renal, or endocrine diseases that could interfere with baseline laboratory parameters.
4. Data Incompleteness: Cases with missing or insufficient preoperative laboratory records.

#### 2.3.4. Study Setting

This retrospective study was conducted at Istanbul Atlas University Hospital, a tertiary referral center providing specialized pediatric cardiology and cardiac surgery services. The diagnosis of congenital heart disease was established by pediatric cardiologists primarily based on transthoracic echocardiographic findings. In patients with complex cardiac anatomy requiring further anatomical clarification, additional imaging modalities such as cardiac catheterization and computed tomography (CT) were utilized.

### 2.4. Data Collection and Hematological Parameters

All laboratory parameters were obtained from blood samples collected during hospital admission in the preoperative period as part of routine clinical evaluation. Patients were routinely evaluated for signs of infection, and those with clinically and hematologically evident infection were excluded according to the study criteria. Clinical and surgical data were retrospectively retrieved from the institutional electronic medical records. The following variables were analyzed:

- Demographic and clinical data: Age, sex, and baseline peripheral oxygen saturation (SpO_2_).
- Comprehensive laboratory data: Availability of complete preoperative hematological and biochemical profiles obtained during hospital admission in the preoperative period as part of routine clinical evaluation, including hemoglobin (Hb), hematocrit (Hct), white blood cell (WBC) count, absolute neutrophil and lymphocyte counts, platelet count, C-reactive protein (CRP), and procalcitonin (PCT).
- Biochemical inflammatory markers: CRP and PCT levels.

#### Calculation of Inflammatory Indices

Systemic inflammatory indices were calculated using the preoperative CBC data as follows:

- NLR: Absolute neutrophil count/absolute lymphocyte count.
- PLR: Absolute platelet count/absolute lymphocyte count.
- SII: (Platelet count × Neutrophil count)/Lymphocyte count.

### 2.5. Statistical Analysis

Statistical analyses were performed using SPSS software (version 26.0, IBM Corp., Armonk, NY, USA). Continuous variables are presented as mean ± standard deviation (SD) or median (interquartile range, IQR), as appropriate, while categorical variables are expressed as frequencies and percentages. The normality of data distribution was assessed using the Shapiro–Wilk test. Variables that did not meet normality assumptions were analyzed using non-parametric tests (Mann–Whitney U test). Comparisons between cyanotic and acyanotic congenital heart disease groups were performed using the independent samples *t*-test or the Mann–Whitney U test, as appropriate. Categorical variables were analyzed using the Chi-square test or Fisher’s exact test. A two-sided *p*-value < 0.05 was considered statistically significant.

Correlation analyses between oxygen saturation and hematological and inflammatory parameters were performed using Pearson or Spearman correlation coefficients, depending on data distribution. Receiver operating characteristic (ROC) curve analysis was performed to evaluate the discriminative ability of inflammatory indices (NLR, PLR, and SII) in distinguishing between cyanotic and acyanotic congenital heart disease. The area under the curve (AUC) and 95% confidence intervals (CI) were calculated.

To account for potential confounding effects, multivariable linear regression analyses were performed to assess the independent association between inflammatory indices and cyanotic status, adjusting for age and body weight.

## 3. Results

A comparative analysis of clinical, hematological, and inflammatory parameters between cyanotic and acyanotic congenital heart disease (CHD) groups is presented in Table 1. Oxygen saturation was significantly lower in the cyanotic group compared with the acyanotic group (75 ± 9% vs. 95 ± 4%, *p* < 0.001). In contrast, hemoglobin and hematocrit levels were significantly higher in cyanotic patients (16.1 ± 2.9 g/dL vs. 13.1 ± 2.0 g/dL and 50.8 ± 9.7% vs. 39.7 ± 5.5%, respectively; *p* < 0.001), reflecting compensatory hematological adaptation to chronic hypoxemia. No significant differences were observed between the groups in white blood cell count or differential leukocyte counts. Due to non-normal distribution, CRP and PCT levels were analyzed using non-parametric methods.

No significant differences were observed between the groups. CRP and procalcitonin levels were presented as median (IQR) due to non-normal distribution and showed no significant differences between groups. Similarly, inflammatory indices derived from complete blood counts, including NLR, PLR, and SII, were comparable between cyanotic and acyanotic patients.

Oxygen saturation was significantly lower in the cyanotic group (75 ± 9% vs. 95 ± 4%, *p* < 0.001). In contrast, hemoglobin and hematocrit levels were significantly higher in cyanotic patients (16.1 ± 2.9 g/dL vs. 13.1 ± 2.0 g/dL and 50.8 ± 9.7% vs. 39.7 ± 5.5%, respectively).

No significant differences were observed between the groups in white blood cell count, differential leukocyte counts, CRP, or PCT levels. Similarly, inflammatory indices derived from complete blood counts, including NLR, PLR, and SII, were comparable between cyanotic and acyanotic patients.

Patients with cyanotic congenital heart disease were categorized into diagnostic subgroups as presented in Table 2. The most frequent subgroup was single-ventricle physiology (31.6%), followed by ventricular septal defect with pulmonary atresia (22.2%) and tetralogy of Fallot (13.9%). Patients with two-ventricle physiology who had previously undergone palliative surgery accounted for 18.4% of the cohort, whereas other diagnoses were less common.

**Figure 1:**
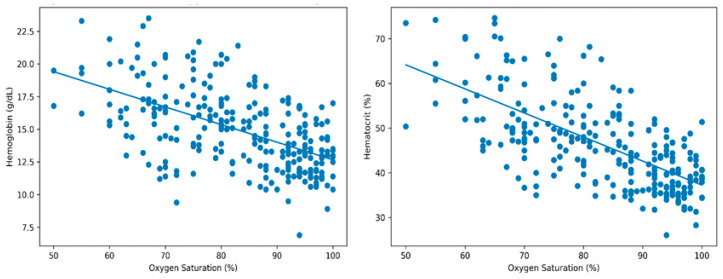
Correlation between oxygen saturation and hematological parameters.

Similarly, inflammatory indices derived from complete blood counts, including NLR, PLR, and SII, were comparable between cyanotic and acyanotic patients (all *p* > 0.05). Correlation analysis demonstrated a moderate negative correlation between oxygen saturation and hemoglobin (r = −0.55, *p* < 0.01) and a stronger negative correlation with hematocrit (r = −0.66, *p* < 0.01), indicating an enhanced erythropoietic response with decreasing oxygen saturation. No significant correlations were observed between oxygen saturation and inflammatory indices (NLR, PLR, and SII) (Figure 1).

ROC curve analysis was performed to assess the discriminative ability of inflammatory indices. All evaluated markers (NLR, PLR, and SII) demonstrated poor discriminative performance, with area under the curve (AUC) values close to 0.5, indicating limited ability to distinguish between cyanotic and acyanotic CHD.

Multivariable linear regression analyses were performed to adjust for potential confounders, including age and body weight. After adjustment, hemoglobin and hematocrit remained significantly associated with group status, whereas inflammatory indices (NLR, PLR, and SII) showed no significant association. These findings indicate that the observed differences in hematological parameters are independent of age and body weight.

## 4. Discussion

This study provides a comprehensive evaluation of hematological parameters and systemic inflammatory indices in pediatric patients with cyanotic and acyanotic congenital heart disease. The principal finding is that chronic hypoxemia in cyanotic CHD is associated with a marked hematological response, characterized by significantly elevated hemoglobin and hematocrit levels alongside reduced oxygen saturation. It should be noted that the inflammatory markers evaluated in this study are indirect and relatively nonspecific. Therefore, the absence of significant differences in these indices does not necessarily exclude the presence of underlying inflammatory processes. Indeed, inflammatory indices, including NLR, PLR, and SII, remained comparable between cyanotic and acyanotic groups. Although age and body weight differed between groups, additional multivariable analyses adjusting for these variables confirmed that the main findings were not influenced by these potential confounders.

These findings were supported by comparative and correlation analyses. Across all analyses, oxygen saturation and erythrocyte-related parameters emerged as dominant discriminative variables, while inflammatory markers demonstrated limited classification value.

Despite the diagnostic heterogeneity of the cyanotic cohort, which was predominantly composed of complex lesions such as single-ventricle physiology and severe conotruncal anomalies, the observed hematological adaptations were remarkably consistent across subgroups. This suggests that compensatory erythropoietic responses represent a shared physiological mechanism in cyanotic CHD. Taken together, these findings suggest a relative dissociation between hypoxemia-driven hematological adaptation and the evaluated systemic inflammatory indices, although this does not exclude the presence of underlying inflammatory processes.

The clinical and hematological differences observed between the two cohorts underscore the profound physiological impact of chronic hypoxemia. The significantly lower age and body weight in the cyanotic group are consistent with the well-established association between cyanotic CHD and impaired somatic growth, likely driven by persistent tissue hypoxia, increased metabolic demand, and altered energy utilization.

Chronic hypoxemia is a potent stimulus for erythropoiesis, leading to elevated hemoglobin and hematocrit levels as a compensatory mechanism to enhance oxygen-carrying capacity. In this context, the higher nucleated red blood cell (NRBC) counts observed in cyanotic patients further reflect intensified bone marrow activity and, in more severe cases, may indicate extramedullary erythropoiesis secondary to sustained hypoxic stress.

From a pathophysiological perspective, complex congenital cardiac lesions characterized by right-to-left shunting result in systemic desaturation and represent the primary drivers of chronic hypoxemia in this population [13]. Importantly, preoperative cyanosis has also been associated with increased early and late postoperative mortality in pediatric cardiac surgery, highlighting its clinical significance beyond baseline physiological adaptation [14].

Defining optimal hemoglobin thresholds in cyanotic children remains clinically challenging [15,16]. Although fixed hemoglobin cutoffs, such as 15 g/dL, have been proposed to define anemia in cyanotic CHD [17], such thresholds may exceed physiological norms for healthy children across different age groups [18,19]. Consequently, the use of uniform hemoglobin thresholds in this population may be misleading.

In cyanotic CHD, the degree of compensatory erythrocytosis is closely linked to resting peripheral oxygen saturation [20]. Therefore, hemoglobin levels considered within normal limits in healthy children may, in fact, represent relative anemia in patients with severe hypoxemia, whose oxygen delivery requirements are substantially increased.

In this context, a saturation-adjusted interpretation of hemoglobin values may be more clinically meaningful than fixed population-based thresholds. Supporting this concept, Zhou et al. [21] demonstrated that a lower preoperative Hb × SpO_2_ value is associated with adverse outcomes in children with cyanotic CHD, highlighting the prognostic relevance of integrating oxygenation status into hemoglobin assessment.

The physiological importance of compensatory erythrocytosis is further underscored by its critical role in preserving tissue oxygenation. Kussman et al. [22] demonstrated that children with cyanotic CHD maintain relatively stable cerebral oxygen saturation despite markedly reduced systemic arterial oxygen levels compared with acyanotic patients. This preservation of cerebral oxygenation is largely attributable to increased hemoglobin concentrations, which enhance oxygen-carrying capacity under hypoxemic conditions.

Consistent with this mechanism, our findings of significantly elevated hemoglobin and hematocrit levels in the cyanotic cohort provide additional clinical evidence supporting this adaptive response. In line with prior studies, a strong inverse relationship between hemoglobin concentration and oxygen saturation has also been described in cyanotic patients, reflecting a compensatory physiological balance [23].

Importantly, hemoglobin levels in pediatric populations are influenced by age-related physiological variation [18,19], further complicating the interpretation of absolute thresholds. Taken together, these findings suggest that compensatory erythrocytosis, accompanied by increased NRBC levels, represents a critical adaptive trade-off that enables the maintenance of adequate tissue oxygen delivery in the setting of chronic systemic hypoxemia.

Interestingly, systemic inflammatory indices did not significantly differ between cyanotic and acyanotic patients in the present study. This finding challenges the intuitive expectation that chronic hypoxemia would be associated with heightened systemic inflammation. Instead, emerging molecular evidence suggests that the dominant biological response to sustained hypoxemia in cyanotic CHD is predominantly metabolic and physiological rather than inflammatory.

For instance, Dong et al. [24] demonstrated that myocardial tissue in cyanotic CHD undergoes substantial metabolic reprogramming under hypoxic conditions, characterized by shifts in energy utilization pathways rather than activation of systemic inflammatory cascades. This adaptive metabolic response, together with compensatory erythrocytosis, may contribute to maintaining physiological homeostasis without triggering a measurable increase in circulating inflammatory markers.

The clinical relevance of inflammatory indices, particularly the neutrophil-to-lymphocyte ratio (NLR), in pediatric cardiac populations remains an area of ongoing debate. In line with our findings, Manuel et al. [7] reported no significant difference in baseline NLR values between cyanotic and acyanotic patients. These observations support the notion that inflammatory indices may have limited value in distinguishing underlying hemodynamic phenotypes.

However, accumulating evidence suggests that NLR may be more informative as a prognostic rather than diagnostic biomarker. Elevated preoperative NLR levels have been associated with adverse postoperative outcomes, including low cardiac output syndrome [8] and increased morbidity [10]. Similarly, higher NLR values have been linked to worse outcomes following staged palliative procedures such as the bidirectional Glenn operation [25,26], and have even been identified as an independent predictor of mortality in high-risk neonatal surgeries, including the Norwood stage I procedure [27].

Taken together, these findings indicate that while systemic inflammatory indices do not differentiate cyanotic from acyanotic CHD at baseline, they retain important prognostic value by identifying patients at increased risk for adverse clinical outcomes.

Correlation analyses in the present study further reinforce the predominance of hematological adaptation. Oxygen saturation demonstrated significant inverse correlations with hemoglobin and hematocrit levels [28,29,30,31], indicating that erythropoietic responses intensify as oxygen saturation declines.

Importantly, this pattern was consistently supported by comparative and correlation analyses. Oxygen saturation and hematological parameters were the primary variables distinguishing cyanotic and acyanotic CHD, whereas inflammatory indices contributed minimally to group differentiation.

### 4.1. Biological Mechanism

The hematological differences observed between cyanotic and acyanotic CHD can be largely attributed to physiological adaptation to chronic hypoxemia. Sustained arterial desaturation activates hypoxia-inducible signaling pathways, particularly the hypoxia-inducible factor (HIF) axis, leading to increased erythropoietin production and subsequent stimulation of erythropoiesis in the bone marrow.

This adaptive response results in elevated hemoglobin and hematocrit levels, enhancing oxygen-carrying capacity and partially compensating for reduced arterial oxygen saturation. In this context, erythrocytosis represents a coordinated and essential physiological mechanism aimed at preserving tissue oxygen delivery under hypoxic conditions.

In contrast, the lack of significant differences in inflammatory indices suggests that chronic hypoxemia predominantly drives hematological and metabolic adaptation rather than systemic inflammatory activation, supporting the concept that these processes are regulated through distinct biological pathways.

### 4.2. Clinical Implications

From a clinical perspective, these findings suggest that conventional hematological parameters provide more meaningful insight into physiological adaptation in cyanotic CHD than novel inflammatory indices. While markers such as NLR, PLR, and SII have demonstrated prognostic value across various cardiovascular conditions, their utility in differentiating cyanotic from acyanotic congenital heart disease appears limited.

Accordingly, inflammatory indices should be interpreted with caution in pediatric CHD populations, as they may not reflect the underlying pathophysiological processes related to chronic hypoxemia. Instead, greater emphasis should be placed on hypoxia-driven erythropoietic responses, which appear to represent the dominant adaptive mechanism in this setting.

### 4.3. Study Strengths and Limitations

This study has several notable strengths. It provides a comprehensive evaluation of both conventional hematological parameters and commonly used inflammatory indices in a pediatric CHD population. In addition, the use of complementary statistical methods, including comparative, correlation, and receiver operating characteristic analyses, allowed for a consistent assessment of the relationship between hematological and inflammatory parameters. Overall, these approaches contributed to the robustness of the findings.

Nevertheless, several limitations should be acknowledged. First, the retrospective single-center design may limit the generalizability of the findings. Second, although the sample size was adequate for the primary analyses, it may not fully capture the heterogeneity of congenital heart disease subtypes. Third, inflammatory biomarkers can be influenced by external factors such as subclinical infection, treatment status, or perioperative conditions, which cannot be completely controlled. Finally, the lack of longitudinal follow-up precluded evaluation of the temporal dynamics and prognostic implications of these biomarkers. The cyanotic congenital heart disease group in this study included a heterogeneous spectrum of diagnoses with differing underlying pathophysiological characteristics and disease severity. Although patients were grouped based on oxygenation status to address the primary study objective, analyzing these diverse conditions as a single group may have obscured potential subtype-specific differences. Future studies with larger and more homogeneous cohorts are needed to explore potential differences among specific cyanotic subgroups.

## 5. Conclusions

In conclusion, children with cyanotic congenital heart disease exhibit a pronounced hematological adaptation to chronic hypoxemia, characterized by elevated hemoglobin and hematocrit levels that serve to preserve tissue oxygen delivery. In contrast, novel inflammatory indices, including NLR, PLR, and SII, do not significantly differ between cyanotic and acyanotic CHD, suggesting a limited role in reflecting baseline disease phenotype.

These findings highlight a relative dissociation between hypoxemia-driven erythropoietic adaptation and systemic inflammatory activation, indicating that the primary biological response to chronic hypoxemia in cyanotic CHD is predominantly hematological, while inflammatory responses were not reflected in the evaluated markers.

Clinically, this underscores the importance of prioritizing oxygenation status and hematological parameters in the assessment of these patients while interpreting inflammatory indices with caution.

Future prospective, multicenter studies with longitudinal follow-up are warranted to further define the prognostic utility of inflammatory biomarkers and their potential role in risk stratification in congenital heart disease. These findings should be interpreted with caution, as they do not exclude the presence of underlying inflammatory mechanisms that may not be captured by the evaluated biomarkers.

## Figures and Tables

**Table 1:** Comparison of clinical, hematological, and inflammatory parameters between cyanotic and acyanotic congenital heart disease.

Parameter	Cyanotic (Mean ± SD)	Acyanotic (Mean ± SD)	*p*-Value
Age (months)	51.37 ± 64.77	75.19 ± 79.25	0.012
Weight (kg)	15.54 ± 15.23	22.71 ± 21.77	0.004
SpO_2_ (%)	75.26 ± 9.43	94.78 ± 3.94	<0.001
WBC (10^3^/µL)	10.92 ± 5.01	11.07 ± 4.45	0.804
Neutrophil (%)	40.28 ± 17.14	46.26 ± 31.72	0.083
Lymphocyte (%)	48.26 ± 16.55	51.01 ± 56.73	0.634
Monocyte (%)	8.62 ± 2.90	8.12 ± 2.53	0.145
Platelet (10^3^/µL)	289.22 ± 102.29	307.48 ± 107.05	0.173
Hemoglobin (g/dL)	16.18 ± 2.94	13.09 ± 2.04	<0.001
Hematocrit (%)	50.82 ± 9.73	39.68 ± 5.50	<0.001
NLR	1.17 ± 1.22	1.47 ± 1.77	0.135
PLR	6.90 ± 4.05	7.69 ± 4.97	0.180
SII	322.68 ± 344.93	403.84 ± 480.81	0.142
IG (%)	0.38 ± 1.08	0.35 ± 0.61	0.773
NRBC (Per 100 WBCs)	0.27 ± 1.03	0.05 ± 0.22	0.008
CRP (mg/L)	2.0 (2.0–5.7)	2.0 (2.0–5.7)	0.596
PCT (ng/mL)	0.02 (0.02–0.06)	0.02 (0.02–0.06)	0.359

Continuous variables are presented as mean ± standard deviation (SD) or median (interquartile range, IQR), as appropriate. *p*-values were calculated using the independent samples *t*-test or Mann–Whitney U test, as appropriate. NRBC, nucleated red blood cells; WBC, white blood cell count; NLR, neutrophil-to-lymphocyte ratio; PLR, platelet-to-lymphocyte ratio; SII, systemic immune–inflammation index; IG, immature granulocytes; CRP, C-reactive protein; PCT, procalcitonin.

**Table 2:** Distribution of cyanotic congenital heart disease subgroups.

Subgroup	*n*	%
Single ventricle physiology	50	31.6
Ventricular septal defect + pulmonary atresia	35	22.2
Tetralogy of Fallot	22	13.9
Two-ventricle physiology with prior palliative surgery	29	18.4
Transposition of the great arteries (TGA)	7	4.4
Double outlet right ventricle + pulmonary stenosis	5	3.2
Intact ventricular septum + pulmonary atresia	3	1.9
Rare anomalies	7	4.4

## Data Availability

The data supporting the findings of this study are not publicly available due to privacy and ethical restrictions but are available from the corresponding author upon reasonable request.
